# Approach to the Fatigue and Cellular Behavior of Superficially Modified Porous Titanium Dental Implants

**DOI:** 10.3390/ma15113903

**Published:** 2022-05-30

**Authors:** Paloma Trueba, Carlos Navarro, Mercè Giner, José A. Rodríguez-Ortiz, María José Montoya-García, Ernesto J. Delgado-Pujol, Luisa M. Rodríguez-Albelo, Yadir Torres

**Affiliations:** 1 Departamento de Ingeniería y Ciencia de los Materiales y del Transporte, Escuela Politécnica Superior, Universidad de Sevilla, 41011 Seville, Spain; ptrueba@us.es (P.T.); jarortiz@us.es (J.A.R.-O.); erndelpuj@alum.us.es (E.J.D.-P.); lralbelo@us.es (L.M.R.-A.); ytorres@us.es (Y.T.); 2 Departamento de Ingeniería Mecánica y Fabricación, Escuela Técnica Superior de Ingeniería, Universidad de Sevilla, 41092 Seville, Spain; cnp@us.es; 3 Departamento de Citología e Histología Normal y Patológica, Universidad de Sevilla, 41009 Sevilla, Spain; 4 Departamento de Medicina, Universidad de Sevilla, 41009 Sevilla, Spain; pmontoya@us.es

**Keywords:** porous dental implant, fatigue resistance, cellular behavior, surface roughness, chemical etching, bioglass coating

## Abstract

In this work, the fatigue and cellular performance of novel superficially treated porous titanium dental implants made up using conventional powder metallurgy and space-holder techniques (30 vol.% and 50 vol.%, both with a spacer size range of 100–200 µm) are evaluated. Before the sintering stage, a specific stage of CNC milling of the screw thread of the implant is used. After the consolidation processing, different surface modifications are performed: chemical etching and bioactive coatings (BG 45S5 and BG 1393). The results are discussed in terms of the effect of the porosity, as well as the surface roughness, chemical composition, and adherence of the coatings on the fatigue resistance and the osteoblast cells’ behavior for the proposed implants. Macro-pores are preferential sites of the nucleation of cracks and bone cell adhesion, and they increase the cellular activity of the implants, but decrease the fatigue life. In conclusion, SH 30 vol.% dental implant chemical etching presents the best bio-functional (in vitro osseointegration) and bio-mechanical (stiffness, yield strength and fatigue life) balance, which could ensure the required characteristics of cortical bone tissue.

## 1. Introduction

Commercially pure titanium (c.p. Ti) and its alloys are among the most widely employed metallic biomaterials for the convenient replacement of damaged cortical bone tissues [1,2], considering their appropriate biocompatibility, corrosion resistance and mechanical strength. However, these materials have two main drawbacks which compromise the clinical success of implants. On one hand, their stiffness (100–110 GPa) is much greater than that of cortical bone (20–25 GPa). This difference can cause the loss of bone and raise the threat of the rupture of the nearby bone [1,3]. Therefore, different methods are proposed to solve these problems. An approach to the reduce the stress-shielding phenomenon and to encourage bone-in-growth into the implant is the introduction of porosity into the implants [3,4]. Biomechanical properties such as the hardness, resistance to corrosion, fracture, fatigue, and wear of implants rely on the amount, volume, and form of the pores, besides the size and geometry of the dental implant. Another way to decrease the stress-shielding phenomenon is the use of β-titanium alloys, as their Young’s modulus (60–80 GPa) is closer to cortical bone´s, and additionally, their biomechanical performances are also improved [2,5,6,7,8,9].

Other drawbacks are the poor osseointegration of the implants, which is inherent in the inert biological behavior of titanium surfaces [10], and the potential infections [11] (proliferation and growth of bacteria) that occur during surgery or the scarring period, which inhibit the formation of new bone. This fact increases the loading times of the dental implant and can cause its uselessness at the medium and long term. In order to address these problems, the literature proposes the manipulation and optimization of the topography (roughness and texture) and the chemistry (bioactive surfaces) of implants, including the immobilization of proteins on the surface [1,11,12,13]. The macro-topography is determined by the geometric design [12] (the presence of threads and conical shapes, etc.), while the surface micro-topography and nano-topography cause effects at the cellular and protein levels, respectively [14].

One of the routes used for this purpose is chemical etching [15], considering its attractive cost, versatility and repeatability. The nature and concentration of the chemical reagent used are particularly important, as are the etching times implemented. On the other hand, among the ways to facilitate the chemical interaction between the implant and cortical bone tissue are the use of bioactive coatings [16,17], thermochemical treatments [18] or the bio-functionalization of the surface [19].

Furthermore, the failure in service of dental implants can also occur due to an overload and/or a poor resistance to fatigue [10,20,21,22]. In this scenario, it is widely known that the accomplishment of successful dental implants depends not only on the quantity/quality of the patient’s bone tissue [23], material used, size, and implant design [24] but also on the surface treatments [25]. In the literature, we found some works in which different routes of manufacture and superficial modification of porous implants have been used. There are also studies that propose computational models and simulations to investigate the stress distribution at the threads, section changes, and pores, etc., or to estimate and understand the static and cyclical behavior in service [26,27,28]. However, few investigations focus on the fatigue behavior of porous dental implants, or the influence of surface modification treatments on their response.

Unfortunately, although enormous advances in this field have been accomplished, dental implants as perfect medical prosthesis devices still remain as an enormous clinical challenge. In this study, the influence of porosity (content, size, and morphology), surface modification treatments (chemical etching and bioactive coating), and fatigue resistance are studied for porous dental implants that were previously obtained via conventional powder metallurgy and space-holder techniques. Finally, exploratory studies of the cellular characterization (the attachment and proliferation of osteoblasts) of new manufactured dental implants are also addressed. The final objective is to propose an implant with a better balance of bio-mechanical (stiffness, yield strength and fatigue limit) and bio-functional (osseointegration and bone ingrowth) performances.

## 2. Materials and Methods

**Figure 1:**
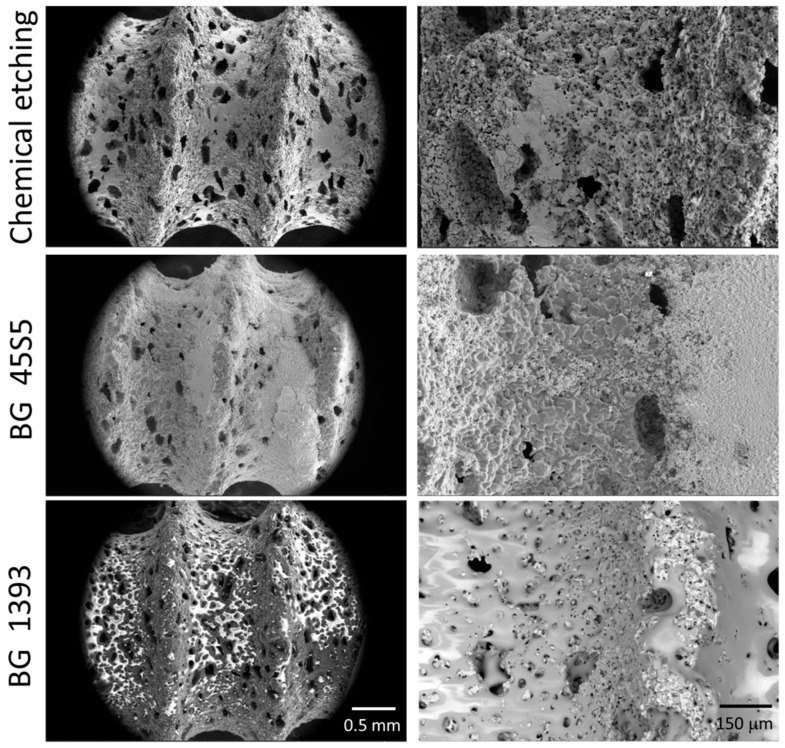
SEM image of the superficially treated porous dental implants.

In this work, the conventional powder metallurgical route (PM) and space-holder technique (SH: ammonium bicarbonate as a spacer–NH_4_HCO_3_) were used for the manufacture of the porous titanium dental implants. The titanium grade IV powder and the spacer particles (30 vol.% and 50 vol.%, both with size range between 100 µm and 200 µm) were mixed in a Turbula^®^ T2C Shaker-Mixer for 40 min to achieve good homogenization. Next, the powder mix was uniaxially compressed in a cylindrical die (8 mm in diameter) at 300 MPa using an Instron 5505 universal testing machine. Later, the green samples were micro-milled using a CNC machine (Roland, Model MDX-40, Shinmiyakoda, Japan) to obtain the thread of the dental implant. Before the sintering step (1250 °C for 2 h, and 10^−5^ mbar), the spacer particles were removed in a conventional oven (60 °C and 110 °C, both for 12 h and 10^−2^ mbar). Furthermore, the surfaces of the dental implants were chemically etched or coated with two bioglasses (BG 45S5 and BG 1393). All of the details of the protocols related to the fabrication and surface modification treatments implemented in this study were used by the authors using first porous titanium disks [15,29] and then similar porous dental implants [30]. In these investigations, the details of the porosity measurements (Archimedes’ method and image analysis) and surface roughness (scanning electron microscopy and confocal laser) of the dental implants used in this investigation can also be consulted. Three of the superficially modified porous implants are shown in Figure 1.

### 2.1. Characterization of the Fatigue Behavior of the Porous Dental Implants Studied

In this section, firstly, the study of the mechanical behavior (static and cyclical) of the virgin porous dental implants (without surface modification) is presented, in order to obtain the influence of the pores (percentage, size, and irregularity). Finally, a preliminary study of the fatigue behavior of superficially treated porous dental implants is shown.

**Figure 2:**
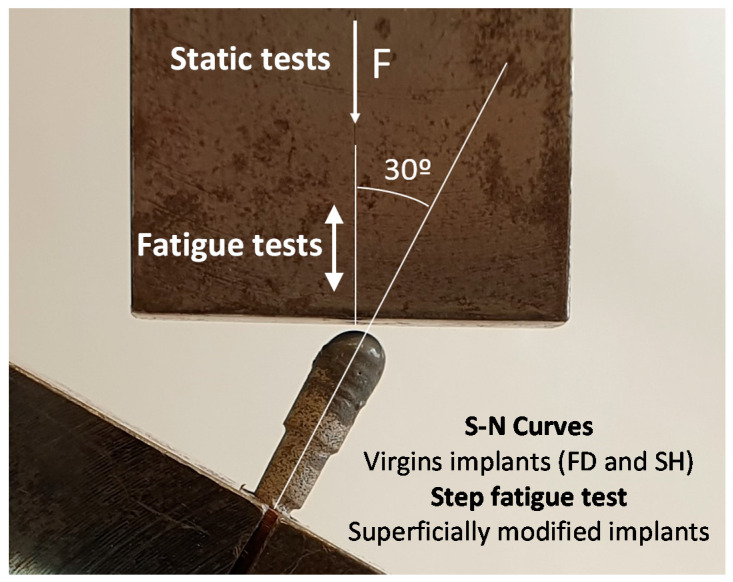
Setup and parameters of the static and fatigue test of the porous dental implants, following the standard ISO 14801.

The mechanical characterization was performed following the test setup proposed in the ISO 14801 standard (see Figure 2) [31]. In these investigations, the load must be applied with an angle of 30° from the axis of the implant, and the piece applying the load was allowed to rotate about the semispherical part of the dental implant. For its part, the fixing plane was placed at a distance of *l* = 11 mm from the center of this semi-sphere, and 3 mm below the plane where the bone level would be, as in a real application. In this work, the first static tests were conducted at a load rate of 10 N/s in order to estimate the loads in the corresponding fatigue tests. On the other hand, the fatigue resistance testing of porous dental implants obtained by PM and SH (30 vol.% and 50 vol.%) routes was carried out at a load ratio of R = 0.1, at a frequency of 15 Hz, and until the complete fracture of the implant. An adequate parameter to compare the mechanical behavior of dental implants is nominal stress, σ, as the sizes of implants are included in this calculation, such that implants with different dimensions could be compared. In order to calculate this stress, the applied force, *F*, can be decomposed into a component generating a compression in the dental implant (*F* ∙ cos 30°) and a component generating a bending moment at the fixing plane of the implant (*F* ∙ sin 30°). The bending moment was obtained by multiplying this force by the distance to the fixing plane, *l*. Only the bending moment will be used to calculate the nominal stress because it was the one generating the tensile stress, which produces the fatigue damage. Assuming, in this case, an implant with a solid circular section with diameter *d* = 3.45 mm, the nominal stress can be calculated using the well-known expression for a circular beam subjected to a bending moment, *M*:

(1) $$\sigma =\frac{32\times M}{\pi \times d^{3}}=\frac{32\times F\times sin30\times l}{\pi \times d^{3}}$$

In this work, seven virgin porous dental implants were tested in order to assess their fatigue resistance. However, considering the additional economic cost of the implemented surface modification treatments (a chemically etched surface or being coated with BG 45S5 or BG 1393), a different fatigue test procedure was performed, to be compared to the conventional tests (S-N curves). In this other test protocol (step fatigue test), the configuration parameters and the load ratio are the same as those used in the conventional test. For the new fatigue test proposed, instead of applying a constant amplitude load, the test starts with a cycle with a maximum load of 40 N, to be later increased by 10% every 50,000 cycles until failure. The fatigue resistance evaluated with the described protocol depends on the potential accumulation of damage in the different previous steps. Then, the results should be rationalized considering this fact. In this context, the maximum stress before failure in this last step is not appropriate to compare the fatigue life with the results of the conventional fatigue tests, because it does not reflect the damage accumulated in the previous steps with lower loads. Therefore, an equivalent constant amplitude stress level that produces the same fatigue damage as in the step test was defined. This equivalent stress can be directly compared to the conventional fatigue tests. In order to achieve this purpose, the concept of fatigue linear damage accumulation was used [32,33]. The fatigue damage was obtained by adding the damage in each load step, with the damage in each step being the ratio between the applied number of cycles, *n_i_*, and the number of cycles to failure, if the corresponding load was the only one applied, *N_i_*. Mathematically, it is possible to calculate the equivalent load that would have to be applied in a constant amplitude load test in order to produce the same damage, *D*, to the implant in the same total number of cycles, *n* (this is the sum of all of the *n_i_*). This is shown in Equation (2):

(2) $$D=\sum \frac{n_{i}}{N_{i}}=\frac{n}{N_{eq}}$$

where *N_i_* and *N_eq_* can be calculated if the fatigue curve of the material is known, *σ ∙ N^b^* *= C.* In Equation (2), *N_eq_* is the number of cycles to failure in a fatigue test in which only the equivalent load is applied. Equation (2) was then transformed into Equations (3) and (4):

(3) $$\sum \frac{n_{i}}{C^{1/b}}\times \sigma_{i}^{1/b}=\frac{n}{C^{1/b}}\times \sigma_{eq}^{1/b}$$

(4) $$\sigma_{eq}={\left(\frac{1}{n}\times \sum n_{i}\times \sigma_{i}^{1/b}\right)}^{b}$$

where *n_i_* and *σ_i_* are the number of cycles and the nominal stress in each segment, respectively, and *n* is the total number of cycles in the test.

Finally, a study of the possible fracture surfaces associated with the monotonic and cyclic tests was carried out by scanning electron microscopy, SEM (Teneo, FEI, Eindhoven, The Netherlands), in order to figure out the origin and the responsible mechanisms of the fracture of the different porous dental implants (with and without surface modification).

### 2.2. Cellular Characterization of Superficially Modified Porous Dental Implants

In this section, the effect of the porosity and surface treatment on the cell behavior of the dental implants studied is addressed.

#### 2.2.1. In Vitro Cell Culture

The MC3T3-E1 mouse pre-osteoblast cell line was grown (CRL-2593 from the American Type Culture Collection (ATCC), Manassas, VA, USA). The implants were sterilized in an autoclave (121 °C, 1.05 kg·cm^−2^, 20 min). We seeded 30,000 cells/cm^2^ above each implant. In order to calculate the number of cells to be seeded, the area of the implant was considered [30]. The cells were grown in Minimum Essential Medium (αMEM) plus 10% fetal bovine serum (FBS) and antibiotics (100 U/mL penicillin and 100 mg/mL streptomycin sulphate) (Invitrogen, Carlsbad, CA, USA), at 37 °C and 5% CO_2_. At 48 h, the medium was changed to osteogenic induction with α-MEM medium, 10% FBS, 10 mM ascorbic acid (Merck, Darmstadt, Germany), and 50 µg/mL β-glycerophosphate (StemCell Technologies, Vancouver, BC, Canada). The medium was changed every 2 days. The in-vitro cell experiments were carried out at 21 days.

#### 2.2.2. Cell Differentiation by Alkaline Phosphatase (ALP) Evaluation

The MC3T3 differentiation levels by alkaline phosphatase (ALP) activity were conveniently evaluated using the Alkaline Phosphatase Assay kit (Colorimetric) (Abcam, Cambridge, UK). All of the determinations were performed in triplicate in order to measure the absorbance at 405 nm of 4-nitrophenol. The data were expressed as U/mL of *p*-nitrophenyl Phosphate (PNPP).

#### 2.2.3. Cell Morphology

After 21 days, the cells were fixed in 10% formalin, followed by a dehydration step using ethanolic solutions (in 30%, 50%, 60%, 70%, 80% and 90% ethanol for 10 min each); then, they were gold-coated using a sputter coater (Pelco 91000, Ted Pella, Redding, CA, USA). The culture was analysed using scanning electron microscopy (SEM) (Zeiss EVO LS 15 scanning electron microscope (Zeiss, Oberkochen, Germany)) with an acceleration voltage of 10 kV.

#### 2.2.4. Statistical Analysis

All of the results are expressed as the mean and standard deviation. The statistical test used was a two-way ANOVA and Tukey’s post-test (SPSS v.22.0 for Windows, IBM Corp., Ar-monk, NY, USA). All of the determinations were analysed in triplicate. *p* ˂ 0.05 was considered statistical difference.

## 3. Results and Discussion

Table 1 shows results of the experimental static behavior (fracture load and nominal stress) of the studied virgin dental implants, together with the collected values of the Young’s (*E_N_*) and dynamic Young’s modulus (*E_d_*, by Nielsen approximation), and the yield strength (*σ_y_*) of the virgin implants, using equations previously described in the literature [34,35,36], which establish the relationships between microstructural parameters (the porosity and morphology of the pores) and their mechanical behavior. Although porous implants have reliable yield-strength values, 200 and 135 MPa for SH 30 vol.% and 50 vol.%, respectively, which are close to the values for cortical bone tissue (150–180 MPa [37,38]), the stiffness (90 GPa) and yield strength (638 MPa) of the conventional PM implants were not satisfactory to find a solution to the stress-shielding phenomenon (20–25 GPa, [37,39]). In this case, it would be necessary to manufacture implants with greater porosity, with the intact structural integrity of the implant during the micro-milling stage and/or under service conditions. In light of this, the decrease in compaction pressure, temperature, and/or sintering time, as well as the use of spacers (included in this study) could arise as possible solutions to cope this problem. Furthermore, although it will be discussed below, it is worthwhile to point out that the gradient of porosity of the SH implants could influence both the stiffness and fatigue behavior between the core and the threads (in contact with the bone) of the implants, to create a gradient of the corresponding Young’s modulus [29]. Finally, focusing on the implant rigidity, the influence of the size and geometry of the implant should also be considered [23,24].

**Figure 3:**
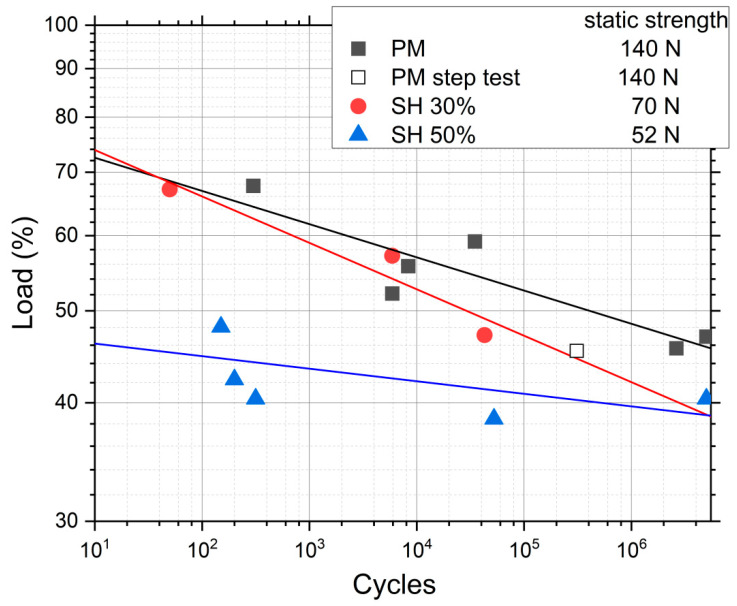
Results of the conventional fatigue tests for implants with different porosities, and the result of one test with the procedure of the step fatigue test. The load percentage is with respect to the static strength.

Figure 3 shows the results of the conventional fatigue tests for PM, SH 30 vol.% and SH 50 vol.%, where the load applied in each test is rationalized with the static strength shown in Table 1. This shows that the PM and SH 30 vol.% have a similar qualitative trend: a fatigue limit between 40% and 50% of the static strength, and a similar fatigue behavior in the rest of the curve. The SH 50 vol.% implant has a different behavior with a slightly lower relative fatigue limit, but a much lower fatigue strength compared to its static strength for the lower lives. It seems that, at this level of porosity, a different fatigue mechanism appears compared to the other two types of implants. The trend lines in Figure 3 represent the statistical fit to the experimental results using the typical fatigue curve mentioned earlier, *σ* · *N^b^* = *C*. The coefficients of regression, *R*^2^, of these fitted curves are 0.79, 0.92 and 0.47 for the implants PM, SH 30 vol.% and SH 50 vol.%, respectively. A very high scatter appears in SH 50 vol.%, which explains the poor fit. Table 2 shows the nominal stresses and the number of cycles in the fatigue tests for the three porous implants studied. An inverse relationship is observed between the porosity of the dental implants (the pore content and size) and the fatigue resistance values obtained for 10^5^ cycles: 100.3 MPa (PM), 44.9 MPa (SH 30 vol.%), and 29.0 MPa (SH 50 vol.%). The fatigue strength at 10^7^ cycles has values of 27–35 MPa and 200–430 MPa for the cortical bone and the commercially pure titanium implants (obtained by a forging process), respectively [40]. In this context, it could be indicated that superficially modified dental implants potentially guarantee the mechanical requirements of the bone tissue to be replaced. An increase in fatigue resistance could even be expected, once the bone in-growth and osseointegration of the implant have occurred.

**Figure 4:**
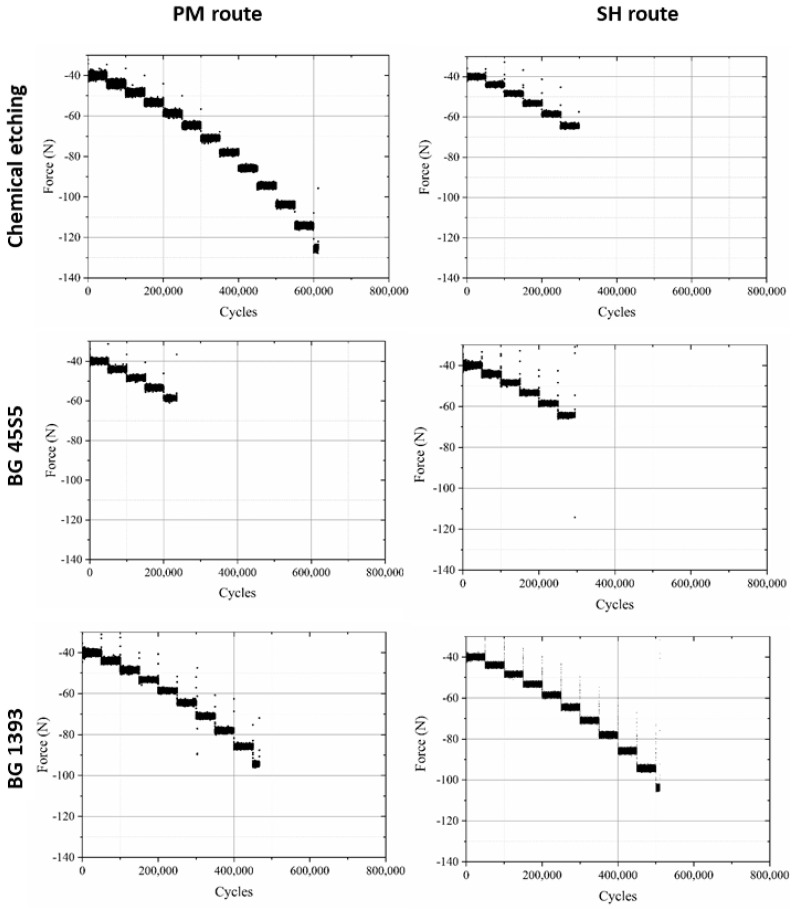
Fatigue behavior of PM and SH (30 vol.%) superficially modified implants (chemical etching, coated with BG 1393 or BG 45S5).

**Figure 5:**
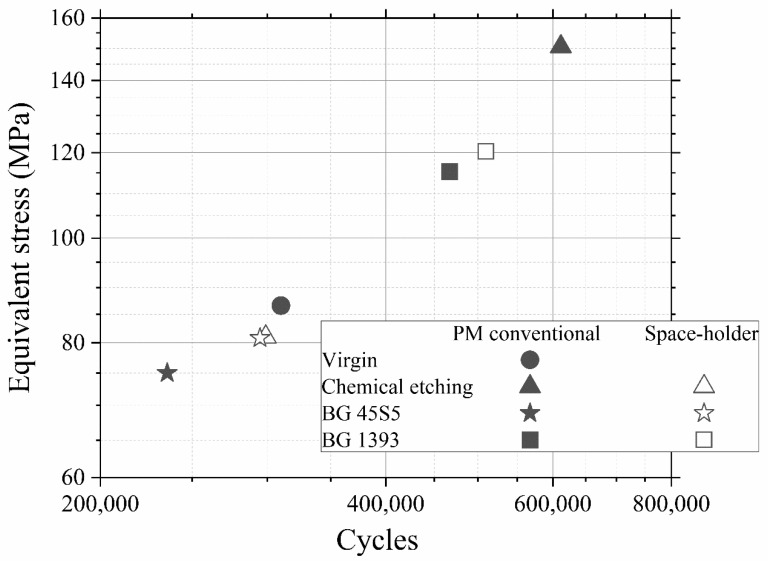
Equivalent stress vs number of cycles. Note: Superficially modified dental implants could guarantee the mechanical requirements (the cortical bone tissue presents a fatigue strength at 10^7^ cycles of 27–35 MPa [40]).

Furthermore, in Figure 3 a data point called “PM step test” is drawn. This point comes from a step fatigue test, as described in Section 2.1, in which the total number of cycles was 310,056 and the load in the last step was 70.9 N. As was also explained in the experimental section, this equivalent stress can be directly compared to the conventional fatigue tests. In this case, the parameters of the fatigue curves are obtained from the conventional fatigue tests already shown, although, as seen in Equation (4), only the slope of the curve, *b*, is needed. The values are *b* = 0.035 for PM and *b* = 0.049 for SH. This particular point matches the data trend obtained in the conventional fatigue tests, assuming the typical scatter in fatigue. Therefore, we can conclude that it is perfectly valid to use the technique of the step fatigue test together with the equivalent stress to analyze and compare different implants with different surface treatments using only one fatigue test. However, the ideal would be a complete fatigue curve; this procedure gives the opportunity to achieve a first discrimination when there is little material available. In this sense, in Figure 4, the maximum loads vs. the number of cycles in step fatigue tests of superficially modified implants are presented. The fatigue behavior showed dependency on the accumulated damage over a certain number of cycles with different load levels. Another semi-quantitative comparison could be made using the nominal stress instead of the applied load. As mentioned earlier, this would be useful in the future in order to compare the results with an implant of distinct size. In this context, the results (equivalent stress, *σ_eq_*, vs. *N*) are shown in Figure 5. This equivalent stress is calculated using Equation (4), where the slope of the fatigue curve is assumed to be the same as in the fatigue tests of the virgin implants. Furthermore, the fatigue curves for these treated implants could be estimated using the points in Figure 5 and the already mentioned slope of the fatigue curve, *b* (0.035 for PM and 0.049 for SH). Table 3 shows the fatigue data of the tests, including the estimated fatigue strength for a fatigue life of 10^5^ cycles, assuming the same fatigue slope as in the virgin implants.

**Figure 6:**
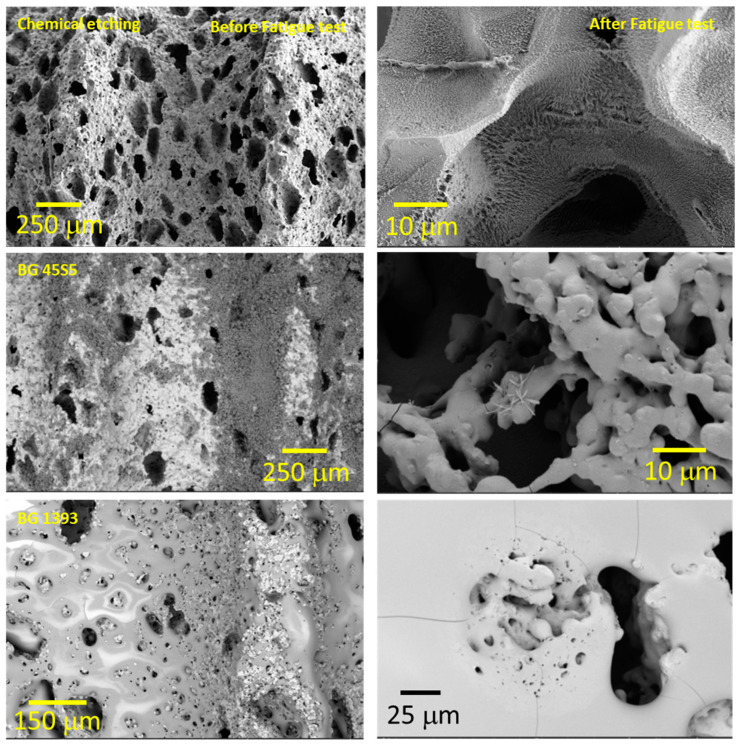
Coated implant surface before and after the fatigue tests (SH: 30 vol. %). Note: Observe the more irregular macro-pores when using chemical etching, and the nucleation of micro cracks in the pores under cyclic loading.

**Figure 7:**
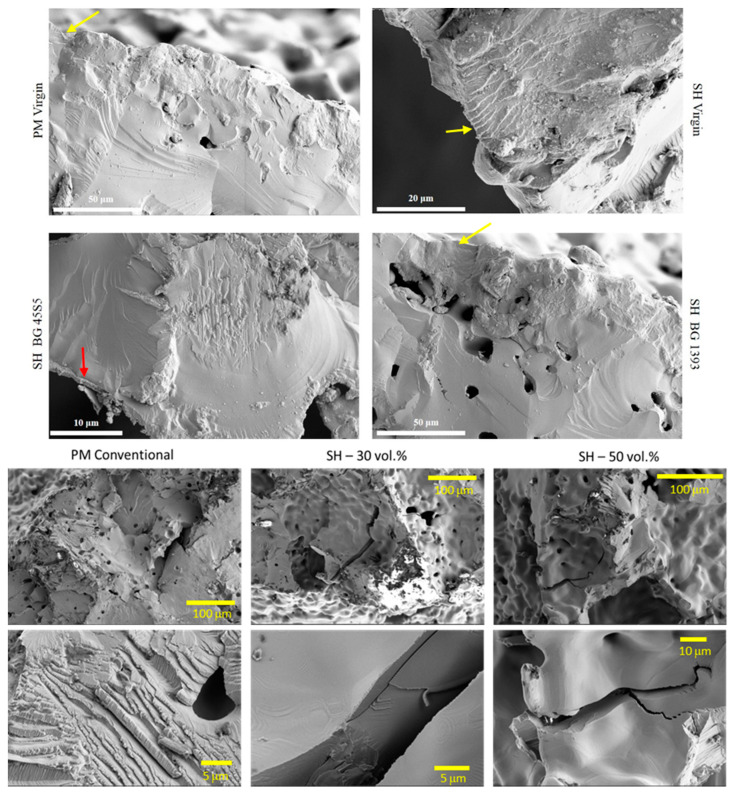
Fracture surfaces under cyclic loads of PM and SH dental implants (virgin and superficially treated), comparing virgin and modified surfaces. Note: Observe the presence of small areas of fatigue striations (see the yellow arrows), and the poor adherence of the BG 45S5 coating (see the red arrow).

The general analysis of these fatigue results allowed (Figure 4, Figure 5 and Figure 6) us to indicate the following aspects:

(i) Modified PM implants: The chemically etched implant and the bioactive glass BG 1393 coated implant presented a higher fatigue resistance than the virgin PM implant, while this was less for the implant coated with BG 45S5. On the other hand, the improvement in the fatigue life of the chemically etched implant may be associated with the formation of a more stable oxide layer on the surface of the implant, usually rutile. This oxide hardened the surface, and thus hindered the movement of dislocations and/or nucleation of micro-cracks under cyclic loads [41]. Comparable results were already reported by Apachitei et al. [42]. They studied in detail the effect of plasma electrolytic oxidation coatings on the fatigue properties of Ti6Al4V and Ti6Al7Nb alloys under physiological conditions (Hank’s solution at 37 °C) in order to describe the fact that oxidized Ti6Al7Nb alloys exhibit an improved fatigue behavior if compared to oxidized Ti6Al4V alloys, independently from the coating thickness. Furthermore, the best fatigue behavior of the implant coated with BG 1393 could be explained by its better adhesion with the Ti implant [43,44] compared to BG 45S5. This fact could be associated with the best compatibility between its thermal expansion coefficients [45]. Furthermore, the temperature used during the coating treatment (exceeding the melting temperature of BG 1393) allowed its infiltration into the macro-pores (see Figure 6).
(ii) Modified SH implants: As previously described, the fatigue behavior of SH virgin implants was conditioned by the role of macro-pores (associated with the use of spacer particles). However, the resistance under cyclical loads of the modified implants clearly depended on what happened on their surface and how it took place. In this context, after chemical etching, the macro-pores were larger and more irregular, justifying the sudden drop in mechanical strength (see Figure 6). Furthermore, the intrinsic micro-porosity of the BG 45S5 coating and its poor adherence (see the red arrow in Figure 7) compromised their use for this type of solicitation. Finally, despite the good infiltration and adherence of BG 1393, the presence of pre-existing microcracks—originating in the macro-pores after the thermal treatment of this coating—could explain its resistance to fatigue (see Figure 6).

It should be noted that the number of cycles that the tested implants resisted in the last step depended on the history of previous fatigue to which each implant was subjected; that is, it depended on the accumulation of damage that was generated on the surface of the material. Previous cyclic solicitations (minor variations in the applied cyclic stresses) may have caused two types of effects:

(1) surface hardening (virgin c.p. Ti implants);
(2) the nucleation and accumulation of damage to the treated surface, chemically or in the interlayer of the coating-implant joint; and
(3) the subcritical growth of pre-existing micro-cracks in the coating.

On the other hand, the fracture surfaces of the studied dental implants are shown in Figure 7. The presence of cleavage was observed, which is the mechanism responsible for the brittle fracture under static mechanical conditions. In this context, it was difficult to identify and measure the size of the defect that caused the fracture under these two types of mechanical solicitation (monotonic and cyclical). However, small areas of subcritical growth (close to the implant surface) could be elucidated, with the presence of fatigue pseudo-striation.

**Figure 8:**
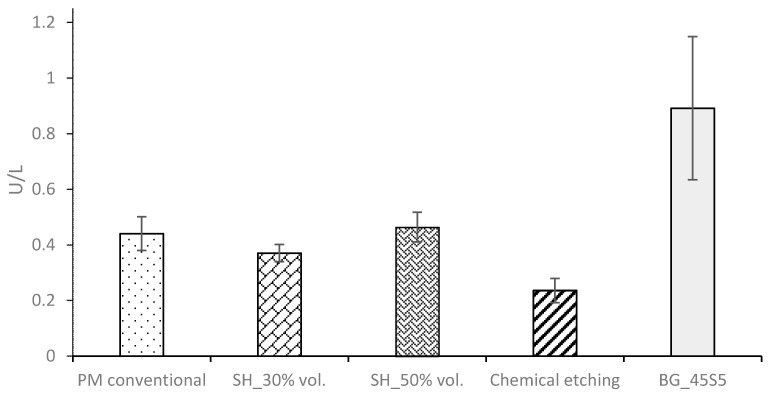
Alkaline phosphatase enzymes (U/mL) in the culture of MC3T3 in different conditions.

**Figure 9:**
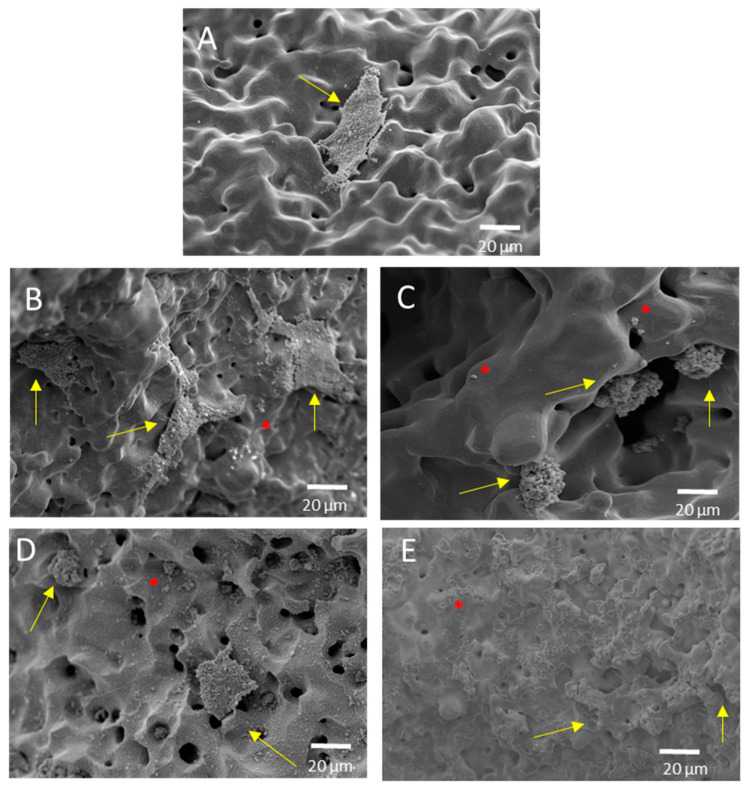
Representative SEM images of the cell adherence in different surfaces of porous dental implants. Virgins: (**A**) PM conventional, (**B**) SH 30 vol. %, (**C**) SH 50 vol. %, and superficially modified. (**D**) Chemical etching and (**E**) BG 45S5, both on conventional PM implants. Note: The cells are indicated in the images (yellow arrow), along with calcium phosphate (red asterisk).

Finally, the ALP activity is used to assess the enhanced in-vitro osseointegration capacity as markers of the early differentiation of osteoblast-like cells. In Figure 8, a general trend of ALP activity is shown, being similar in all the samples; additionally, osteoblast cells have presented an activity of around 2.5 U/L. However, the BG 45S5 implants show the highest cellular activity, although no significant differences were found among the other implants. The higher activity of ALP indicates that they are cultures with more differentiated cells [46], such that the osteoblasts grown on the BG 45S5 implants present greater differentiation and—it is expected—more hydroxyapatite deposits, as was observed on the SEM images (Figure 9). SEM was used to observe the spreading of the MC3T3-E1 cells on each sample. In Figure 9, the presence of osteoblasts cells could be observed on the surface (marked with yellow arrows). Osteoblasts have a triangular shape, which is typical of differentiated cells, with pseudopod protrusion and microfilament extension. Its plasma membrane presents a surface full of sector vesicles, which is indicative of good enzymatic functional activity. In addition, small precipitates with a morphology like hydroxyapatite (marked with red asterisks) are appreciated. However, a future study of XRD should be carried out in order to corroborate this fact. An in vivo test will be performed in the future to validate the reliability of the results. However, their behavior is promising, considering previous results reported in the literature for this type of surface [46].

## 4. Conclusions

The influences of the porosity and surface treatments of dental implants manufactured using PM and SH routes on their fatigue and cellular behavior have provided the following conclusions:

(1) The virgin SH dental implants have a lower fatigue resistance than those obtained by the conventional PM route. The macro-pores control the crack nucleation process, although they can also hinder the propagation of cracks (stop-hole mechanism—the tip of the crack is blunted). On the other hand, the roughness of the walls of these implants favors the adhesion of osteoblasts. Furthermore, an increase in the behavior (ALP activity or cells differentiation) of the in vitro cell cultures is observed after the surface modifications, and the differences between the treatments used are not statistically significant.
(2) The high micro-porosity of the BG 45S5 coating compromised the fatigue behavior of the implant, being 17% less than the value corresponding to PM dental implants without surface treatment. In the case of SH 30 vol.% implants, it also decreased by 65% compared to the virgin implant. On the other hand, the fatigue resistance of conventional PM implants coated with BG 1393 improves by 25%. This increase may be related to the improved infiltration and/or better thermal compatibility (coefficients of expansion) between Ti and the BG 1393. Finally, the increase of the fatigue resistance of the superficially chemically etched porous dental implant (38% vs. PM virgin) is related to the formation of a hard layer of titanium oxide formed during the chemical treatment of the surface.

In summary, SH 30 vol.% the chemical etching of dental implants presents the best bio-functional (in vitro osseointegration) and bio-mechanical (stiffness, yield strength and fatigue life) balance, which could guarantee the requirements of cortical bone tissue (*E* = 20–25 GPa, *Ò_y_* = 150–180 MPa, and *K_Ic_* = 3.5 MNm^−3/2^).

## Figures and Tables

**Table 1:** Static behavior of the virgin porous dental implants.

		Fracture Load (N)	Nominal Stress (MPa)	Estimated Mechanical Behavior [34,35,36]		
				*E_N_* (GPa)	*E_d_* (GPa)	*σ_y_* (MPa)
PM		140 ± 3	191 ± 1	90.9 ± 0.5	86.2 ± 0.6	638 ± 5
SH	30 vol.%	70 ± 4	95.5 ± 1.5	44.6 ± 0.9	45.8 ± 1.0	200 ± 8
	50 vol.%	52 ± 6	71 ± 2	30.3 ± 1.1	35.6 ± 1.0	135 ± 14

Note: The static behavior of superficially modified dental implants is similar to the corresponding virgin implant.

**Table 2:** Nominal stresses and the number of cycles in the fatigue tests.

PM		SH 30 vol.%		SH 50 vol.%	
Cycles	MPa	Cycles	MPa	CYCLES	MPa
300	129.3	50	64.1	150	34.1
34,820	113.0	5900	54.6	200	30.0
8324	106.4	42,855	45.0	52,403	27.3
5900	99.6	-	-	315	28.7
310,056 *	86.6 *	-	-	5 × 10^6^	28.7
5 × 10^6^	89.7	-	-		
2.6 × 10^6^	87.2	-	-		

Note: * Step fatigue test for the PM implant.

**Table 3:** Step fatigue tests of porous dental implants (PM and SH 30 vol.%).

Porous Dental Implants		Maximum Fatigue Load (N)	Nominal Stress (MPa)	Equivalent Maximum Stress (MPa) (See Figure 5)	Number of Total Cycles	Estimated Fatigue Strength at 10^5^ Cycles (MPa)
Virgin	PM	70.9	96.7	86.6	310,056	90.1
Chemical Etching	PM	114.1	155.7	150.5	611,850	160.4
	SH	64.4	87.9	81	298,754	85.5
BG 45S5	PM	58.6	79.9	75	235,260	77.3
	SH	64.4	87.9	80.8	294,670	85.2
BG 1393	PM	94.3	128.7	115.3	466,920	121.7
	SH	103.7	141.5	120.3	510,240	130.3

## Data Availability

Not applicable.
